# Hydroxyhexylitaconic acids as potent IMP-type metallo-β-lactamase inhibitors for controlling carbapenem resistance in *Enterobacterales*

**DOI:** 10.1128/spectrum.02344-23

**Published:** 2024-02-05

**Authors:** Jun-ichi Wachino, Wanchun Jin, Chihiro Norizuki, Kouji Kimura, Motonori Tsuji, Hiromasa Kurosaki, Yoshichika Arakawa

**Affiliations:** 1 Department of Medical Technology, Faculty of Medical Sciences, Shubun University, Ichinomiya, Aichi, Japan; 2 Department of Bacteriology, Nagoya University Graduate School of Medicine, Nagoya, Aichi, Japan; 3 College of Pharmacy, Kinjo Gakuin University, Nagoya, Aichi, Japan; 4 Institute of Molecular Function, Misato, Saitama, Japan; Universidad de Buenos Aires, Buenos Aires, Argentina

**Keywords:** metallo-β-lactamase, *Enterobacterales*, inhibitors

## Abstract

Metallo-β-lactamases (MBLs) represent one of the main causes of carbapenem resistance in the order *Enterobacterales*. To combat MBL-producing carbapenem-resistant *Enterobacterales*, the development of MBL inhibitors can restore carbapenem efficacy for such resistant bacteria. Microbial natural products are a promising source of attractive seed compounds for the development of antimicrobial agents. Here, we report that hydroxyhexylitaconic acids (HHIAs) produced by a member of the genus *Aspergillus* can suppress carbapenem resistance conferred by MBLs, particularly IMP (imipenemase)-type MBLs. HHIAs were found to be competitive inhibitors with micromolar orders of magnitude against IMP-1 and showed weak inhibitory activity toward VIM-2, while no inhibitory activity against NDM-1 was observed despite the high dosage. The elongated methylene chains of HHIAs seem to play a crucial role in exerting inhibitory activity because itaconic acid, a structural analog without long methylene chains, did not show inhibitory activity against IMP-1. The addition of HHIAs restored meropenem and imipenem efficacy to satisfactory clinical levels against IMP-type MBL-producing *Escherichia coli* and *Klebsiella pneumoniae* clinical isolates. Unlike EDTA and Aspergillomarasmine A, HHIAs did not cause the loss of zinc ions from the active site, resulting in the structural instability of MBLs. X-ray crystallography and *in silico* docking simulation analyses revealed that two neighboring carboxylates of HHIAs coordinated with two zinc ions in the active sites of VIM-2 and IMP-1, which formed a key interaction observed in MBL inhibitors. Our results indicated that HHIAs are promising for initiating the design of potent inhibitors of IMP-type MBLs.

**IMPORTANCE:**

The number and type of metallo-β-lactamase (MΒL) are increasing over time. Carbapenem resistance conferred by MΒL is a significant threat to our antibiotic regimen, and the development of MΒL inhibitors is urgently required to restore carbapenem efficacy. Microbial natural products have served as important sources for developing antimicrobial agents targeting pathogenic bacteria since the discovery of antibiotics in the mid-20th century. MΒL inhibitors derived from microbial natural products are still rare compared to those derived from chemical compound libraries. Hydroxyhexylitaconic acids (HHIAs) produced by members of the genus *Aspergillus* have potent inhibitory activity against clinically relevant IMP-type MBL. HHIAs may be good lead compounds for the development of MBL inhibitors applicable for controlling carbapenem resistance in IMP-type MBL-producing *Enterobacterales*.

## INTRODUCTION

Metallo-β-lactamases, one of the common carbapenemases that spread among *Enterobacterales*, are a clinical obstacle toward carbapenem treatment because they can efficiently hydrolyze various β-lactams including carbapenems (1, 2). Therefore, overcoming the problems posed by metallo-β-lactamase (MΒL) is a key therapeutic strategy to revive and maintain the anti-bacterial activity of carbapenems for the treatment of MBL-producing organisms (3). To achieve this, the development of clinically available MΒL inhibitors has been a topic of great research interest, although no MBL inhibitors have been introduced practically in clinical settings so far (4, 5).

Regarding MBL inhibitors, only two agents, namely taniborbactam (VNRX-5513) (phase 3) (6, 7) and xeruborbactam (QPX7728) (phase 1) (8, 9), have been evaluated in clinical trials for their effectiveness. These compounds are dual effectors against both serine-carbapenemases and MBLs. As an inhibitor targeting MBL alone, ANT2681, developed by Antabio, is currently in the preclinical phase of testing (10). These small-molecule MBL inhibitors originated from chemical libraries or were chemically synthesized and further modified to enhance the inhibitory activity toward MBLs together with optimizing ADME (absorption-distribution-metabolism-excretion) profiles.

Previously, we screened for MBL inhibitor candidates targeting IMP-1 MBL and identified the seed of the MBL inhibitor, 2,5-dimethyl-4-sulfamoylfuran-3-carboxylic acid (SFC), which is the preferred inhibitor for IMP-1 [inhibition constant (*K*_*i*_), 0.22 µM] rather than NDM-1 (*K*_*i*_, 9.8 µM). This eventually resulted in the synthesis of a broad range of MBL inhibitor, 2,5-diethyl-1-methyl-4-sulfamoylpyrrole-3-carboxylic acid (11). SFC would not be identified if we initially targeted the NDM-1, instead of the IMP-1. This indicates that screening work targeting IMP-type MBLs enables the identification of inhibitor candidates that would be overlooked when targeting NDM-type MBLs alone.

**Fig 1:**
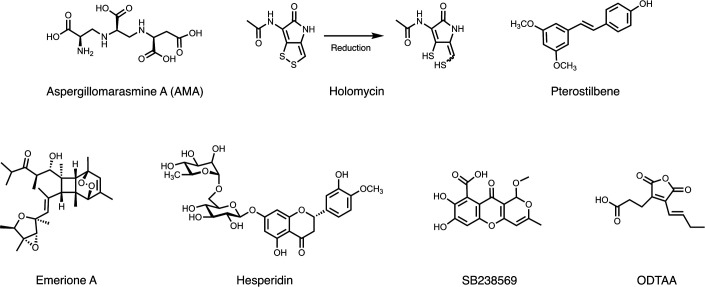
Natural products like metallo-β-lactamase inhibitors.

Microbial natural products have served as important sources of antimicrobial agents targeting pathogenic bacteria since the discovery of antibiotics in the mid-20th century (12, 13). Representative MBL inhibitors derived from natural products are shown in Fig. 1. The most potent agent among them is Aspergillomarasamine A, an extract from *Aspergillus versicolor*, which shows inhibitory activities against NDM- and VIM-type MBLs by chelating their central zinc ions (14, 15). Reduced holomycin has a high affinity toward zinc and showed potent inhibitory activity against NDM-1 (16). Pterostilbene (17), Emerione A (18), and Hesperidin (19) are also natural products that show inhibitory activity against NDM-type MBLs. These three products were identified when targeting NDM-type MBLs in screening studies. Furthermore, inhibitor screening work targeting secondary-relevant IMP-type has rarely been performed; SB238569 was reported as a weak inhibitor of IMP-1 20 years ago (20), and ODTAA extracted from *Paecilomyces* sp. was recently identified as an IMP-1 inhibitor (21). In this study, we screened microbial natural product libraries sourced mainly from *Actinomycetales* and filamentous fungi, targeting IMP-1, and successfully identified hydroxyhexylitaconic acids as superior IMP-type MBL inhibitors.

## RESULTS AND DISCUSSION

### Screening of IMP-1 inhibitors

A total of 5,488 microbial samples were initially subjected to cell-based screening, and 16 samples showed positive results. Among the 16 samples, five inhibited the imipenem (IPM)-hydrolyzing activity of IMP-1 with a residual activity of <50% in an *in vitro* enzyme-based assay. Finally, two samples (F2400 and F1765), with the highest inhibitory activities, residual activity of <30% in enzyme-based assays, and meropenem (MPM) minimum inhibitory concentration (MIC) reduction from 1 to ≤0.03 µg/mL (≥32-fold) in cell-based assays under a certain amount of sample, were subjected to further analyses. In this study, we focused on the F2400 sample prepared from *Aspergillus* sp. strain OPMF00815.

To identify the components responsible for the IMP-1 inhibitory activity of extracts of *Aspergillus* sp. OPMF00815, the extracts were preliminarily fractionated using high-performance liquid chromatography (HPLC) and subjected to enzyme- and cell-based assays. Chromatograms of the extracts from *Aspergillus* sp. OPMF00815 is shown in Fig. S1A and two apparent peaks (A and B) were observed. The fractionated samples corresponding to these two peaks demonstrated apparent IMP-1 inhibitory activity in both enzyme- and cell-based assays. The components corresponding to these two peaks were subjected to preliminary mass spectrometry (MS) analysis, and these showed almost the same profiles with major signals of approximately *m*/*z* at 185, 229, and 481 (Fig. S1B and C). The compounds corresponding to peaks A and B might be structural analogs with very similar chemical structures, although these two components were not completely separated at this point.

### Identification of hydroxyhexylitaconic acids as IMP-1 inhibitor

**Fig 2:**
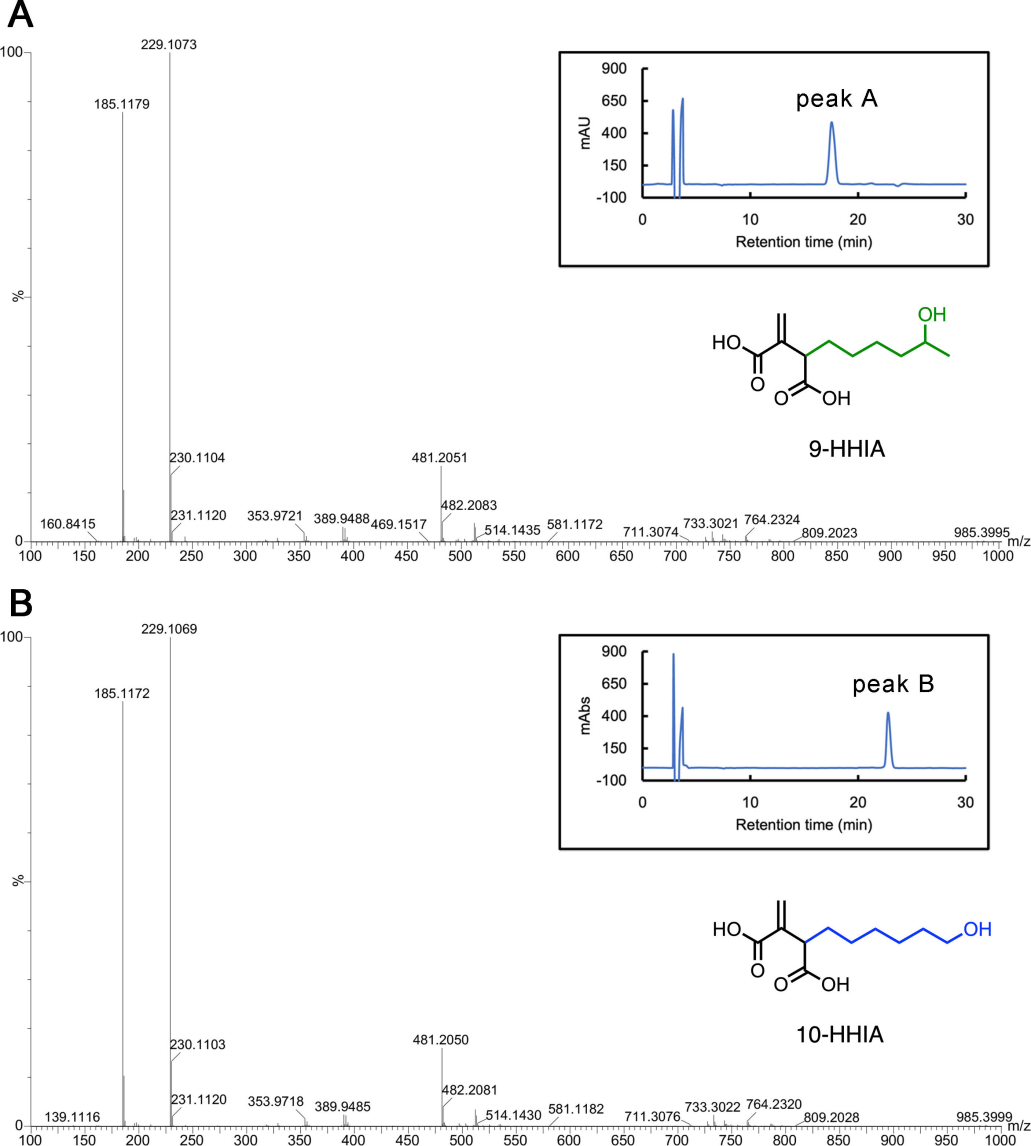
(**A**) MS spectrum of compound A (9-HHIA) showing IMP-1 inhibitory activity. The HPLC profile of purified compound A (9-HHIA) is shown on the upper right side. The chemical structure of 9-HHIA is shown on the lower right side. (**B**) MS spectrum of compound B (10-HHIA) showing IMP-1 inhibitory activity. The HPLC profile of purified compound B (10-HHIA) is shown on the right side. The chemical structure of 10-HHIA is shown on the lower right side.

We performed large-scale production of IMP-1 inhibitory components (corresponding to peaks A and B) by *Aspergillus* sp. OPMF00815 and finally succeeded in fractionating the peak A and peak B components with high purity through the repeated purification step using HPLC (Fig. 2A and B). The MS spectrum of the peak A component showed the presence of a compound with a 229.1073 [M-H]^−^ monoisotopic mass (corresponding to C_11_H_17_O_5_, 229.1076) and C_11_H_18_O_5_ molecular formula (Fig. 2A). This compound (namely compound A) was found to have carboxyl groups owing to the presence of adduct ion peaks (185.1179 [M-H]^−^) corresponding to decarboxylated anions (Fig. 2A). In addition, MS spectrum and molecular formula information indicated that compound A had one hydroxyl group and two carboxyl groups. The ^13^C and ^1^H NMR spectra of compound A are shown in Fig. S2A and S3A, and the summary of the ^1^H NMR results is described in Text S1. In the ^13^C NMR spectrum, the two signals at low magnetic fields (δ_C_ 169.5 and 177.1) indicated the presence of two carbonyl groups corresponding to the MS spectrum and molecular formula of compound A (Fig. 2A). The presence of alkene was supported by the δ_C_ 127.0 and 140.9 signals (Fig. S2A). ^1^H NMR spectrum indicated the presence of one methyl group [*δ*_*H*_ 1.12 (H10)], four methylenes [*δ*_*H*_ 1.46–1.26 (H6, 7, 8), 1.70–1.62 (H5), and 1.87–1.81 (H5)], two methines [*δ*_*H*_ 3.43 (H2) and 3.71–3.64 (H9)], and one exo-methylene proton [*δ*_*H*_ 5.74 (H11) and 6.31 (H11)] (Fig. S3A). Considering the above results, compound A was determined to be 9-hydroxyhexylitaconic acid (9-HHIA). The MS and NMR spectra of 9-HHIA we obtained were almost identical to those of 9-HHIA extracted from *Aspergillus niger* S17-5 strain (22).

The MS spectrum of compound B was almost the same as that of 9-HHIA (Fig. 2A and B), whereas the NMR spectral patterns of compound B differed slightly from those of 9-HHIA (Fig. S2A and B; Fig. S3A and B), indicating that compound B was a structural analog of 9-HHIA. Compound B was identified as 10-hydroxyhexylitaconic acid (10-HHIA). The MS and NMR spectra of 10-HHIA we obtained were similar to those of 10-HHIA isolated by Sano et al. (22). The 9-HHIA and 10-HHIA compounds were itaconic acid (IA) derivatives with a C-C double bond and two carboxyl groups. It has been reported that IA is produced by a variety of microbes, including *Aspergillus* spp., and has been found to have a wide range of bioactivities including anti-bacterial, anti-inflammatory, and anti-cancer activities (23–26). IA is also produced in mammalian macrophages and plays an important role as an anti-inflammatory and anti-bacterial metabolite (25, 27, 28). Furthermore, IA produced by *Aspergillus* spp. has been used in a variety of industrial applications as starting materials for synthetic polymers (29), surfactants (30, 31), and food additives (32). In addition, IA derivatives such as 9-HHIA and 10-HHIA have also been produced by microbes, particularly *Aspergillus* spp., and have demonstrated anti-bacterial, anti-inflammatory, and cytotoxic activities (22). Our results are the first to show that IA derivatives 9-HHIA and 10-HHIA exhibit inhibitory activities toward IMP-1 MBL.

### Taxonomic position of HHIA-producing *Aspergillus* sp. strain OPMF00815

The 9-HHIA- and 10-HHIA-producing *Aspergillus* sp. OPMF00815 analyzed here was isolated from sediment in the estuary of the Nakama River in Okinawa Prefecture, Japan. The strain showed black colonies on lignocellulose agar (LCA) plates, which were similar to those of a member of black *Aspergilli* (Fig. S4A) (33). In the phylogenetic tree based on ITS, β-tubulin, and calmodulin sequences of *Aspergillus niger* clade, *Aspergillus* sp. OPMF00815 was located close to *Aspergillus tubingensis* strain CBS 134.48^T^ (34), which is identified as an industrially important black *Aspergilli* and produces polyketide pigments such as naphtho-gamma-pyrones (35). The average nucleotide identity of *Aspergillus* sp. OPMF00815 and *A. tubingensis* CBS 134.48^T^ was 95.48%. These results indicate that *Aspergillus* sp. OPMF00815 was genetically most close to *A. tubingensis* CBS 134.48^T^ among the investigated *Aspergillus niger* clade.

### Inhibitory activities of 9-HHIA and 10-HHIA against MBLs

**Fig 3:**
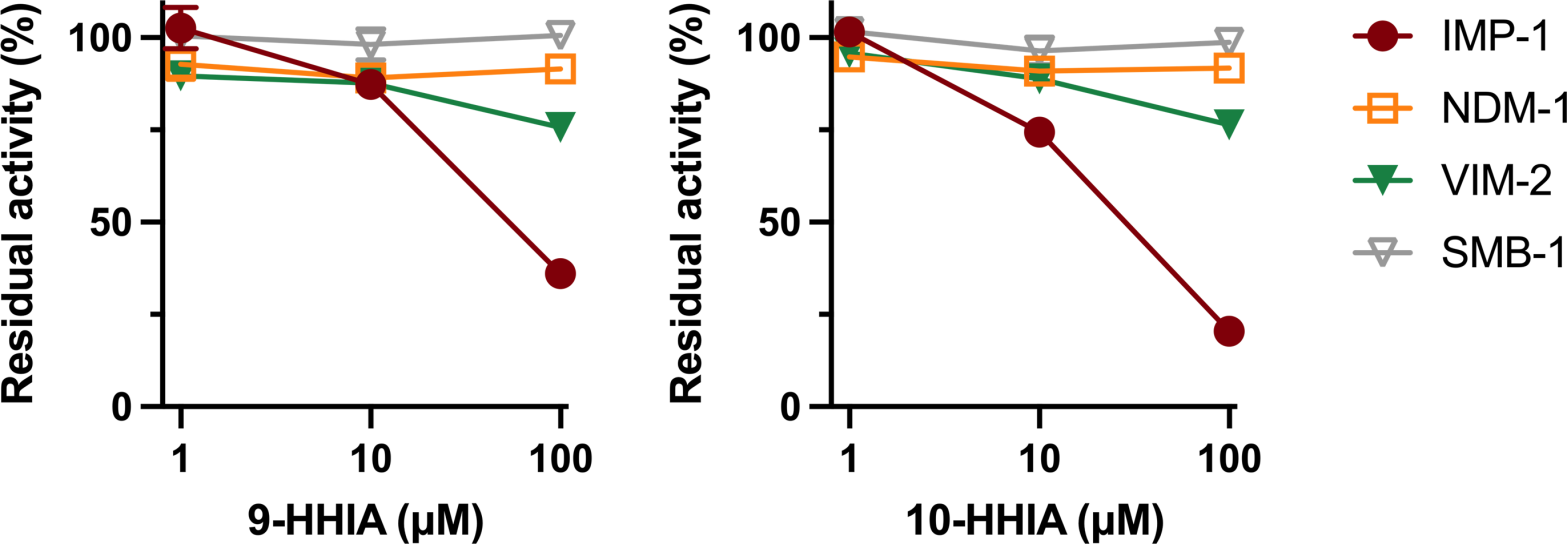
Inhibitory activities of 9-HHIA and 10-HHIA against a variety of MBLs (IMP-1, NDM-1, VIM-2, and SMB-1). Data are the means ± standard deviations of results from three replicates.

We investigated the inhibitory activity of 9-HHIA and 10-HHIA against MBLs, IMP-1 (subclass B1), NDM-1 (subclass B1), VIM-2 (subclass B1), and SMB-1 (subclass B3). The results of *in vitro* inhibition assays are shown in Fig. 3. Both 9-HHIA and 10-HHIA inhibited the IPM-hydrolyzing activity of IMP-1 in a dose-dependent manner. Their inhibitory activities against VIM-2 were moderate, and they did not inhibit the hydrolyzing activity of NDM-1 and SMB-1, even at high doses (100 µM). The results of susceptibility tests for MBL-producing *E. coli* transformants are shown in Table 1 and were consistent with those of *in vitro* enzyme assays. The MICs of MPM for IMP-1-producing *E. coli* were clearly reduced by 9-HHIA and 10-HHIA in a dose-dependent manner (Table 1). On the other hand, the addition of HHIAs moderately reduced ceftazidime MICs for VIM-2-producing *E. coli* but did not reduce the MPM MICs for NDM-1- and SMB-1-producing *E. coli* (Table 1). These findings suggest that the 9-HHIA and 10-HHIA act as selective inhibitors of IMP-1 MBL. IA, which lacked a long methylene chain (a backbone structure colored in black in Fig. 2), did not reduce β-lactam MICs for such MBL producers (Table 1). These results indicate that the elongated methylene groups in 9-HHIA and 10-HHIA are key to exerting their inhibitory activities toward IMP-1.

**TABLE 1:** Results of susceptibility tests for *E. coli* transformants producing MBLs^*a*^

Agents	Concentration (μg/mL)	*E. coli* DH5α			
		pBC-IMP-1	pBC-NDM-1	pBC-VIM-2	pCL-SMB-1
		MIC (μg/mL)			
		MPM	MPM	CAZ	MPM
Control	–	1	64–>64	4–8	32
9-HHIA	6.25	0.5	64	4	32
	25	0.25	64	2	32
	100	0.125	64	2	32
10-HHIA	6.25	0.5	64	4	32
	25	0.125	64	4	32
	100	≤0.0625	64	2	32
Itaconic acid	6.25	1	>64	8	32
	25	1	>64	8	32
	100	1	>64	8	32

^*a*^ MPM, meropenem and CAZ, ceftazidime.

### *In vitro* activity of HHIAs against MBL-producing *Enterobacterales* clinical isolates

The synergistic effects of MPM and 9-HHIA or 10-HHIA were investigated for IMP-, NDM-, and VIM MBL-producing *Enterobacterales* clinical isolates. MPM MIC values for IMP-type (IMP-1, IMP-4, and IMP-6) MBL-producing *Enterobacterales* clinical isolates (*E. coli*, *Klebsiella pneumoniae*, *Klebsiella oxytoca*, *Proteus penneri*, and *Enterobacter cloacae* complex) were reduced in the presence of 9-HHIA or 10-HHIA in a dose-dependent manner (Table 2). At a high concentration of 10-HHIA (100 µg/mL), MPM MICs for eight IMP-type MBL producers were categorized within the susceptible criteria (MPM MIC, ≤1 µg/mL) following the guidelines of the Clinical and Laboratory Standards Institute (36). In contrast, neither 9-HHIA nor 10-HHIA reduced the MPM MIC values for NDM-type and VIM-type MBL-producing strains even at high concentrations. The IPM MICs were also reduced in the presence of HHIAs for IMP-type producers, but the same was not observed for the producers of NDM and VIM types (Table S1). The preferred inhibitory behaviors of HHIAs against IMP-type MBL found in *in vitro* assays were reproduced with the results of susceptibility testing of MBL-producing *Enterobacterales* clinical isolates.

**TABLE 2:** Results of susceptibility tests for MBL-producing *Enterobacterales* isolates

		MPM MIC (μg/mL)						
Bacterial strains	MBLs	Control	+9-HHIA			+10-HHIA		
			(6.25)^*a*^	(25)^*a*^	(100)^*a*^	(6.25)^*a*^	(25)^*a*^	(100)^*a*^
*E. coli* NUBL-22	IMP-6	32	16	16	4	16	8	1
*E. coli* NUBL-24	IMP-1	8	4	0.5	0.125	4	0.5	0.125
*E. coli* 426	IMP-1	16	8	4	0.25	8	1	0.25
*E. coli* 465	IMP-1	32	32	8	0.5	16	4	0.25
*K. pneumoniae* NUBL-8	IMP-1	16	16	4	0.5	8	2	0.25
*K. pneumoniae* AR0034	IMP-4	8	8	2	0.25	4	0.5	0.125
*K. pneumoniae* NUBL-23	IMP-6	64	32	16	0.5	32	2	0.25
*K. oxytoca* NUBL-827	IMP-1	32	32	16	4	16	8	1
*P. penneri* E11-M475	IMP-1	>64	>64	>64	32	>64	>64	16
*E. cloacae* NUBL-5	IMP-1	16	16	16	8	16	16	8
*K. pneumoniae* MS5674	NDM-1/VIM-1	>64	>64	>64	64	>64	>64	64
*K. pneumoniae* AR0076	VIM-1	32	32	32	32	32	32	32

^*a*^ μg/mL.

**Fig 4:**
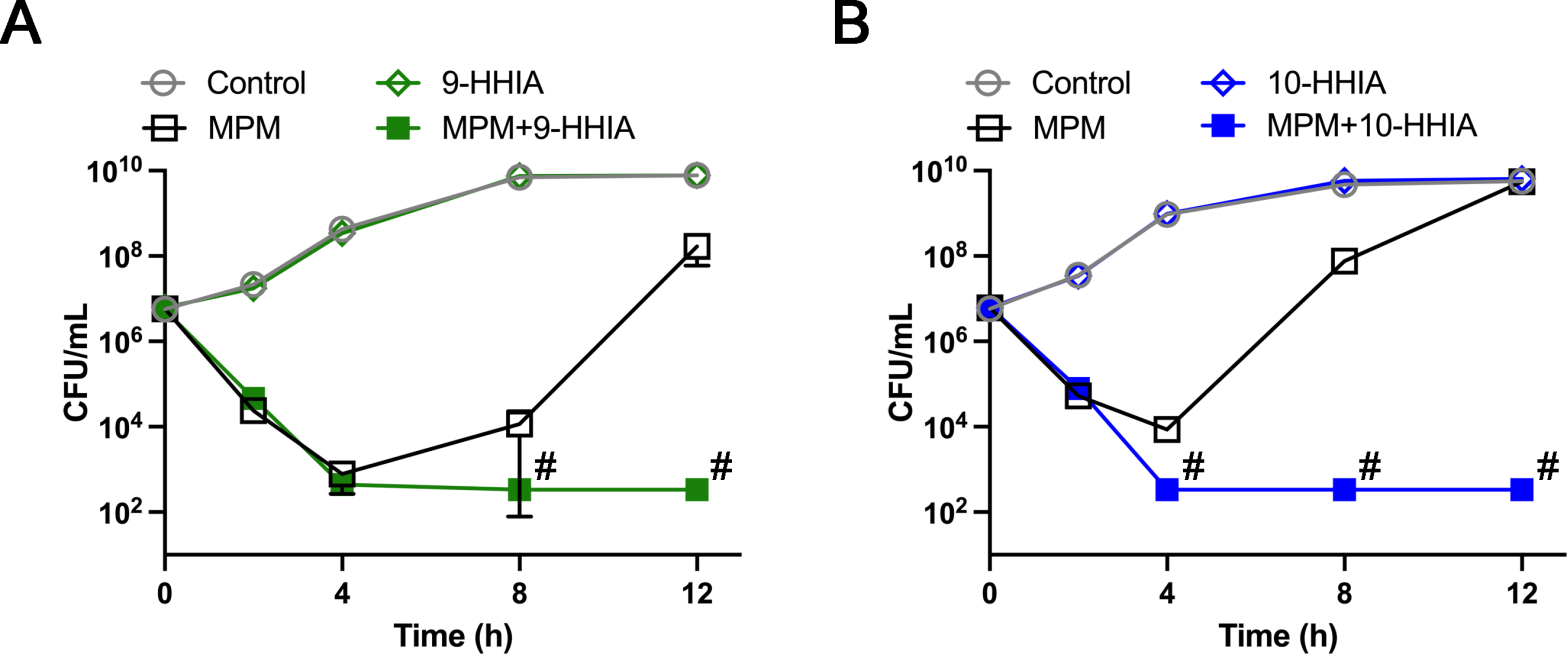
Time-kill curve assays in the presence of MPM or HHIAs alone [(**A**) 9-HHIA and (**B**) 10-HHIA] or their combination during a 12-h incubation. Samples were collected at 0, 2, 4, 8, and 12 h to determine the viable IMP-1-producing *E. coli* NUBL-24 strain numbers. Values are the means of three replicates. The “#” symbol indicates a value less than the detection limit (333 cfu/mL).

Time-kill curves showed that 9-HHIA (50 µg/mL) and 10-HHIA (50 µg/mL) did not affect the growth of IMP-1-producing *E. coli* clinical isolate NUBL-24 (Fig. 4A and B). The addition of MPM (1 µg/mL) reduced the population of live bacterial cells for up to 4 h, but then, regrew close to the control level at 12 h (Fig. 4A and B). The MPM-HHIA combination treatment did not permit the re-growth of bacterial cells (Fig. 4A and B). Our data revealed that the HHIAs (50 µg/mL) could restore the efficacy of meropenem against IMP-1 MBL-producing *E. coli* where it had previously shown almost no original growth inhibitory effect, thereby suppressing the appearance of MPM-resistant bacterial populations.

### Inhibitory mechanisms of HHIAs against IMP-1

**Fig 5:**
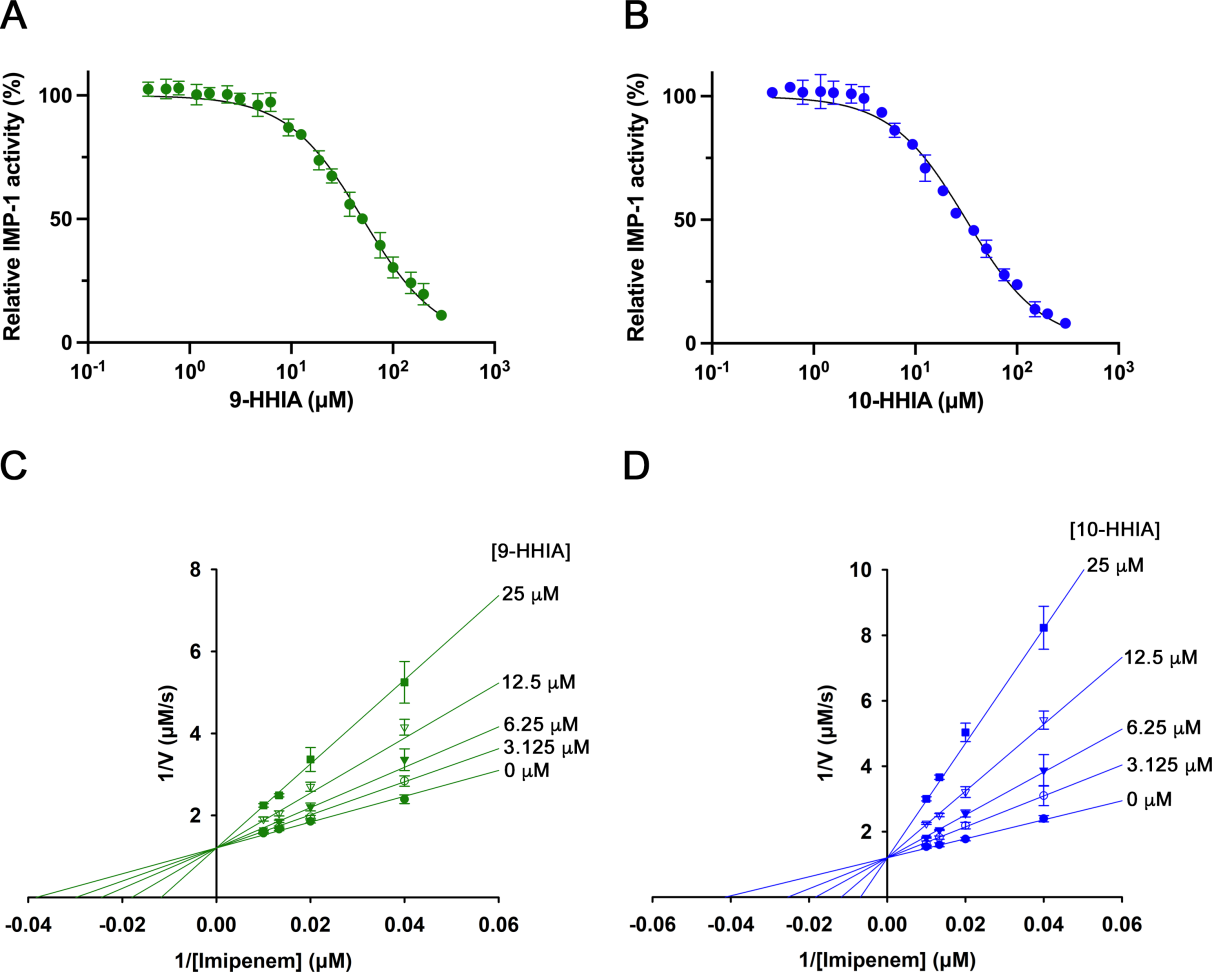
IC_50_ measurements of 9-HHIA (**A**) and 10-HHIA (**B**) for IMP-1. Lineweaver–Burk plot for IMP-1. Lineweaver–Burk plots show competitive inhibition modes for both 9-HHIA (**C**) and 10-HHIA (**D**). Data represent mean ± standard deviations of results from three replicates.

9-HHIA and 10-HHIA had a greater inhibitory effect on IMP-1 than NDM-1 and VIM-2. Therefore, in this study, we determined the inhibitory effects of 9-HHIA and 10-HHIA on IMP-1. The IC_50_ values of 9-HHIA and 10-HHIA for IMP-1 were 50.5 and 31.6 µM, respectively (Fig. 5A and B). To evaluate the inhibition mechanisms of 9-HHIA and 10-HHIA, we determined the *K*_*i*_ values of 9-HHIA and 10-HHIA for IMP-1. Lineweaver–Burk plots showed that both 9-HHIA and 10-HHIA behaved as competitive inhibitors, with micromole-level *K*_*i*_ values of 11.1 and 5.0 µM, respectively (Fig. 5C and D). The 9-HHIA and 10-HHIA molecules also inhibited IMP-6, which has an amino acid substitution at position 262 in contrast to IMP-1, with *K*_*i*_ values of 27.8 and 21.0 µM, respectively.

**Fig 6:**
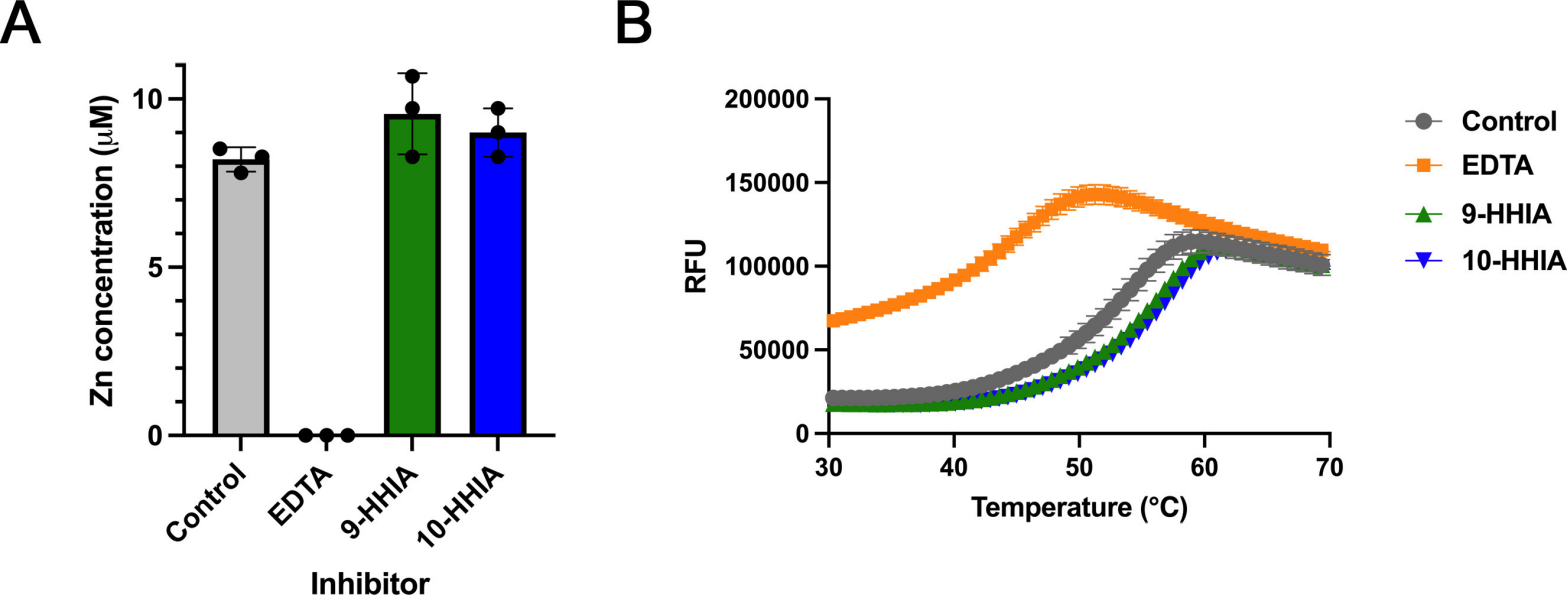
(**A**) Detachment of Zn from the active site of IMP-1 in the presence of EDTA, 9-HHIA, or 10-HHIA. Data represent mean ± standard deviations (SD) of results from three replicates. (**B**) Differential scanning fluorimetry analysis of IMP-1 treated with EDTA, 9-HHIA, and 10-HHIA. Melting curves for IMP-1 alone (gray), with EDTA (orange), 9-HHIA (green), and 10-HHIA (blue). Data represent mean ± SD of results from four replicates.

We investigated the final process of HHIA’s inhibitory action against IMP-1, i.e., whether or not the 9-HHIA and 10-HHIA caused the dissociation of zinc ions from the active site of IMP-1, leading to protein destabilization. First, we performed a zinc content experiment, and the results are shown in Fig. 6A. The zinc ions in IMP-1 were completely lost after EDTA treatment compared to IMP-1, which did not undergo treatment. 9-HHIA and 10-HHIA treatment did not cause a significant loss of zinc ions in IMP-1, and the extent of zinc ions was at the same level as that of the untreated sample. Unlike EDTA, both 9-HHIA and 10-HHIA did not strip zinc ions from the active site.

Differential scanning fluorimetry (DSF) analysis was performed to investigate the structural stability of the inhibitor-treated proteins and the results are shown in Fig. 6B. The structural stability of IMP-1 was largely lost in the presence of excess EDTA, and the melting temperature (*T*_*m*_) of the EDTA-treated protein was 43.4°C ± 0.2°C, which was significantly lower than that of the control protein (52.5°C ± 0.1°C) (Fig. 6B). EDTA dissociated zinc ions from the protein and led to structural instability, which is in accordance with the results of the zinc content experiments. In contrast, the *T*_*m*_ values of IMP-1 treated with 9-HHIA (54.9°C ± 0.1°C) or 10-HHIA (55.6°C ± 0.1°C) were slightly higher than those of the control protein, thus maintaining some protein stability (Fig. 6B). The 9-HHIA and 10-HHIA did not lead to the dissociation of zinc ions from the active site (Fig. 6A) nor increase protein structural instability (Fig. 6B).

These results suggest that the final process of the inhibitory action of HHIAs is different from that of EDTA; the former does not strip zinc ions from the active site, and the latter causes the dissociation of zinc ions. However, dipicolinic acid and 4-(3-aminophenyl) pyridine-2,6-dicarboxylic acid, which are MBL inhibitors carrying two carboxylates as well as HHIAs, stripped zinc ions from the active site of MBLs, in addition to forming ternary complexes (37). It has been revealed that the extent of zinc stripping of MBLs largely depends on the experimental conditions used. Thus, the possibility of the removal of zinc ions by HHIAs cannot be ruled out. The extent of zinc stripping by HHIAs appeared to be extremely low compared to that by EDTA.

### Structural insights into the recognition of 10-HHIA by VIM-2

**Fig 7:**
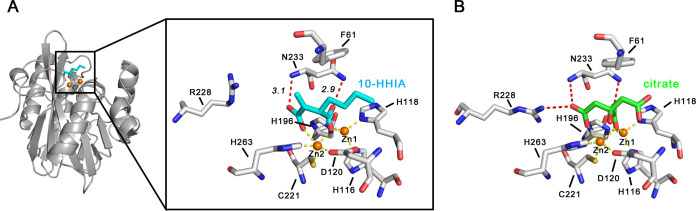
(**A**) Schematic representation of the overall structure of VIM-2 complexed with 10-HHIA and interactions between VIM-2 and 10-HHIA. The 10-HHIA molecule is illustrated in cyan (carbon) and red (oxygen). Zinc ions are presented as orange spheres. The amino acids of VIM-2 are indicated by silver sticks. Yellow and red dashed lines indicate coordination and hydrogen bonds, respectively. (**B**) Interaction modes between VIM-4 and citrate. The citrate is illustrated in green sticks. The amino acids, zinc ions, and bonding are shown as described in panel A. The figure was rendered by PDB data 2WHG. All the figures were drawn using PyMOL (40).

The results of the inhibitory mechanism analyses suggested a stable binding situation between IMP-1 and HHIAs. Therefore, we attempted to reveal the binding mode between IMP-1 and 10-HHIA in detail using X-ray crystallographic analyses. However, we did not obtain cocrystals of IMP-1–10-HHIA, which is suitable for collecting diffraction data. The microseeding technique produced crystals when using IMP-1 solution alone [DMSO (dimethyl sulfoxide) concentration, 1.25%], but crystals did not appear when the IMP-1 solution was mixed with 10-HHIA (DMSO concentration, 1.25%). The IMP-1 single crystals cracked within 15 min when they were soaked in the reservoir solution containing 10-HHIA, and we could not collect the diffraction data of the crystals. Instead, we obtained co-crystals of the VIM-2–10-HHIA complex and collected 1.6 Å diffraction data. The overall structure of the VIM-2–10-HHIA complex is shown in Fig. 7A. One 10-HHIA molecule with clear electron density binds to two zinc ions in the active site of the protein (Fig. S5). The details of the binding mode between VIM-2 and 10-HHIA are enlarged, wherein the carboxylate oxygen O1 coordinates with Zn1 (2.1 Å) and Zn2 (2.1 Å), at which the hydroxyl anion for a nucleophilic attack toward the carbonyl carbon of β-lactams was originally located in the apo-protein structure. The carboxylate oxygen (O2) of 10-HHIA formed hydrogen bonds with the nitrogen atom of the Asn233 side chain (2.9 Å, Fig. 7A) (amino acid positions were assigned with the BBL number). The O3 atom of another carboxylate coordinate Zn2 (2.3 Å, Fig. S5) and the O4 of that bind to the main chain amide of Asn233 (3.1 Å, Fig. 7A) in the protein backbone. The binding of the basic residue Arg228 to 10-HHIA was not identified in this study, although this interaction has been identified as having a significant role in the recognition of substrate β-lactams and inhibitors of VIM-type MBLs (11, 38). The binding mode between VIM-4 and citrate determined by Lassaux et al. is shown in Figure. 7B (39) to compare the central coordination of VIM-2–10-HHIA complex and VIM-4–10-citrate complex (PDB ID: 2WHG). The amino acid identities between VIM-2 and VIM-4 are approximately 91% and the amino acids in the active site, which are required to recognize substrate β-lactams and inhibitors, are included in them. The spatial positions of the two carboxylates of citrate were quite similar to those of 10-HHIA, resulting in a similar binding mode, except for the Arg228-carboxylate interaction. The role of Arg228 in recognizing 10-HHIA is controversial. The side chain shift of Arg228 in VIM-2 original crystals might not occur with the addition of 10-HHIA in our experimental condition. The elongated methylene chain of 10-HHIA was exposed to the solvent side of the protein and appears to have no specific interactions, consistent with the fact that the electron density map corresponding to the top of the methylene chain was not visible (Fig. S5).

### *In silico* docking simulation shows the binding mode between IMP-1 and 10-HHIA

**Fig 8:**
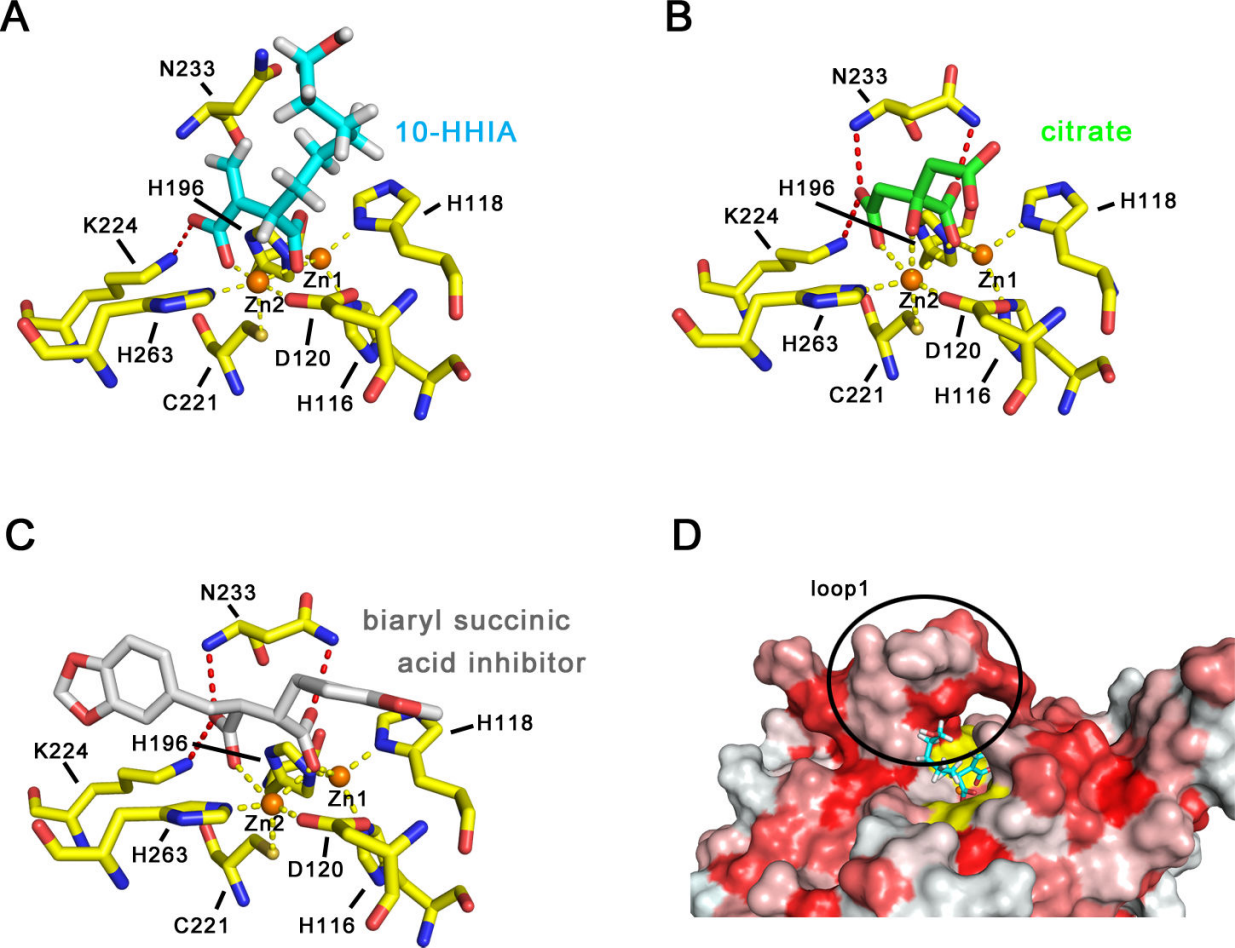
(**A**) The results of *in silico* docking simulation showing binding mode between IMP-1 and 10-HHIA. The 10-HHIA molecule is illustrated in cyan (carbon) and red (oxygen). Zinc ions are presented as orange spheres. The amino acids of IMP-1 are shown in yellow sticks. Yellow and red dashed lines indicate coordination and hydrogen bonds, respectively. (**B**) Interactions between IMP-1 and citrate. The citrate is illustrated in green sticks. Other illustrations were the same as for panel A. The figure was rendered by PDB data 7DTM. (**C**) Interactions between IMP-1 and biaryl succinic acid inhibitor. The biaryl succinic acid inhibitor is illustrated in silver sticks. Other illustrations were the same as for panel A. The figure was rendered by PDB data 1JJE. (**D**) Representation of hydrophobic surfaces of the simulated IMP-1–10-HHIA complex. The redder residues indicate the ones with higher hydrophobicity. All the figures were drawn using PyMOL (40).

We performed an *in silico* docking simulation to obtain the most stable docking mode between IMP-1 and 10-HHIA. The 10-HHIA molecule was prepared in an *S*-enantiomer form based on the VIM-2–10-HHIA complex (Fig. 7A). The most stable docking mode between IMP-1 and (*S*)10-HHIA is shown in Fig. 8A. As seen in the VIM-2–10-HHIA complex, two carboxylates coordinated with the zinc ions at a distance of 1.7 or 1.8 Å. Such coordination around the zinc ions of IMP-1−(*S*)10-HHIA complex was almost the same as that of IMP-1−citrate (Fig. 8B) and IMP-1−biaryl succinic acid inhibitor complexes (Fig. 8C), both of which carry two neighboring carboxylates coordinating the zinc ions (41, 42). Furthermore, 3,6-disubstituted phthalic acid derivatives (43) and disodium 2,3-diethylmaleate (ME1071) (44), comprising the two neighboring carboxylates, were also preferred inhibitors against IMP-type MBLs. These two adjacent carboxylate groups are likely essential for exerting specific inhibitory activity toward IMP-type MBL.

In all three complexes, IMP-1−(*S*)10-HHIA, IMP-1−citrate, and IMP-1−biaryl succinic acid inhibitor, one carboxylate oxygen binds to the basic residue Lys224. However, the involvement of Asn233 in the recognition of (*S*)10-HHIA was not observed, whereas it was observed in both IMP-1−citrate (Fig. 8B) and IMP-1−biaryl succinic acid inhibitor complexes (Fig. 8C). No relationship between Asn233 and (*S*)10-HHIA may be dependent on our apo-IMP-1 crystal structure (PDB ID: 5Y5B) used for the simulation, in which Asn233 originally flipped away from the central active site. Thus, we cannot deny the possibility that Asn233 is involved in the recognition of (*S*)10-HHIA. X-ray crystallographic analysis of IMP-1−(*S*)10-HHIA complex, instead of simulation, may reveal the actual role of Asn233. However, such an analysis was not successful in the present work.

The elongated methylene chain of (*S*)10-HHIA was positioned along the hydrophobic surfaces of the amino acids in the loop1 region (Fig. 8D). The hydrophobic interactions between the methylene chain and loop1 region may enhance the inhibitory activity of (*S*)10-HHIA toward IMP-1, which was supported by the fact that IA without a long methylene chain did not inhibit IMP-1 activity (IC_50_ > 1 mM). Optimization of the methylene side chain of (*S*)10-HHIA would lead to the formation of a compound with higher inhibitory activity than (*S*)10-HHIA. However, a remaining issue of this study is to elucidate the mechanism by which HHIA molecules demonstrated more preferable inhibitory activities toward IMP-type MBLs rather than NDM and VIM types. Unfortunately, we could not completely clarify this mechanism through the structural data presented. Further research would be required to understand the mechanism of selective inhibitory nature toward MBLs.

### Conclusions

Here, we report that HHIAs, such as 9-HHIA and 10-HHIA, produced by a member of the genus *Aspergillus* are potent inhibitors of clinically relevant IMP-type MBLs, rather than NDM and VIM types. Microbial natural products are still useful sources for identifying new MBL inhibitors, and the type of discovered inhibitor would be largely diverse depending on the type of targeted MBL. Clinically relevant and horizontally spreading MBLs, IMP type, NDM type, and VIM type, belong to the same subclass B1 MBL group, and their overall structures and central active site architecture around zinc ions were quite similar to each other. However, there are diversities in local architecture such as loop1 structures, which result in different responses to the inhibitors. Therefore, one approach to identify novel lead compounds that could be optimized as MBL inhibitors in the future would be the differential screening of divergent MBLs.

HHIAs, together with 3,6-disubstituted phthalic acid derivatives and ME1071, which commonly carry two neighboring carboxylates needed for coordinating zinc ions, are the preferred inhibitors of IMP-1. Such inhibitors are good lead compounds for the development of effective inhibitors to control IMP-type MBL-producing organisms and can be structurally extended to cover all clinically relevant MBLs through chemical modifications. In addition, the preferred inhibitory nature of HHIAs toward IMP-type MBLs would apply to the development of methods for the detection and discrimination of IMP-type MBL-producing *Enterobacterales* in clinical microbiology laboratories.

## MATERIALS AND METHODS

### Bacterial strains and recombinant proteins

The bacterial strains used in this study, MBL-producing *E. coli* transformants and *Enterobacterales*, have been listed in our previous reports (11, 45). The expression and purification of IMP-1, IMP-6, NDM-1, VIM-2, and SMB-1 were performed according to previously described methods (11, 45, 46).

### Production and purification of IMP-1 inhibitory components in *Aspergillus* sp. strain OPMF00815

*Aspergillus* sp. OPMF00815, grown on LCA agar plates, was inoculated in modified potato dextrose broth (mPDB) (PDB with KNO_3_, KH_2_PO_4_, Mg_2_SO_4_, and artificial sea water) and incubated at 28°C for 7 days at 250 rpm. Aliquots of pre-cultured cells were further inoculated into fresh mPDB and incubated at 28°C for 7 days at 250 rpm. The culture medium was centrifuged at 2,850 × *g* for 10 min. The supernatant of the culture medium was extracted with ethyl acetate, condensed, and fractionated through a stepwise gradient of methanol (0%, 20%, 40%, 60%, 80%, and 100%) using a Purif-Pack column (ODS, size: 20) (Shoko Science). After stepwise purification with methanol, the samples were repeatedly purified using an HPLC (Shimadzu) equipped with a COSMOSIL 5C18-AR-II packed column (φ20 × 250 mm) (Nacalai Tesque). The mobile phase consisted of solvent A (0.1% formic acid) and solvent B (0.1% formic acid-acetonitrile), and the isocratic elution with 17% solvent B was performed at a flow rate of 10 mL/min and monitored at 210 nm. The cell pellets were treated with acetone and methanol and further treated with ethyl acetate as described above. The samples treated with ethyl acetate were fractionated through a stepwise gradient of methanol and subjected to repeated purification by HPLC, as described above.

### Structural characterization of IMP-1 inhibitory compounds

UPLC-MS (ultra-performance liquid chromatography-mass spectrometry) analysis was performed using an ACQUITY UPLC (Waters) equipped with an ACQUITY UPLC BEH C18 column (φ2.1 × 50 mm) (Waters) and Xevo G2-S QTOF systems (Waters). The mobile phase consisted of solvent A (0.1% formic acid) and solvent B (0.1% formic acid-acetonitrile). The elution was performed with an increase in the gradient from 5% to 100% of solvent B for 0–5 min. The elution rate was 0.8 mL/min. The NMR spectra of the samples were recorded using a JNM-ECZ-600 spectrometer (600 MHz, Jasco). Chemical shifts are reported in parts per million (ppm) on a *δ* scale referring to the residual solvent peak (^1^H, 3.31 ppm, ^13^C, 49.0 ppm for CD_3_OD). The coupling constants (*J*) are reported in Hertz.

### Susceptibility testing

Susceptibility testing, based on the microdilution method, was performed according to the guidelines of the Clinical and Laboratory Standards Institute (36).

### Time-killing assays

The initial density of IMP-1-producing *E. coli* NUBL-24 (MPM MIC, 8 µg/mL) was adjusted to approximately 5 × 10^6^ colony-forming units (cfu)/mL. Either MPM (1 µg/mL) or 9-HHIA/10-HHIA (50 µg/mL) alone or a combination of the two was added to the bacterial solution, and then incubation was performed at 37°C. LB broth containing bacteria alone was used as a control. At 0, 2, 4, 8, and 12 h after adding the agents, an aliquot of the bacterial culture was removed, diluted, and spotted on LB agar plates to count the viable bacterial cells. The detection limit was set at 333 cfu/mL.

### *In vitro* inhibition assay

MBLs were incubated with HHIAs in 20 mM HEPES buffer (pH 7.5) containing 200 mM NaCl and 50 µg/mL BSA at 30°C for 5 min. IPM was added at a concentration of 100 µM, and the rate of IPM hydrolysis was monitored at 297 nm. The initial velocity (*v*_0_) rates were plotted against HHIA’s logarithmic concentrations, and the data were fitted to a four-parameter variable slope to obtain the half-maximal inhibitor concentration (IC_50_) values using GraphPad Prism 9 software (GraphPad Software). The inhibition constant (*K*_*i*_) was determined as follows: *v*_0_ was measured after varying the concentrations of IPM and HHIAs, and inverse *v*_0_ (1*/*_v0_) was plotted against the inverse IPM concentration [1/(IPM)]. Lineweaver–Burk plots were created to obtain *K*_*i*_ values under the competitive inhibition model using the SigmaPlot 14 suite (Hulinks).

### Differential scanning fluorimetry assay

The DSF assay was performed using the Protein Thermal Shift Dye Kit (Thermo Fisher Scientific) and StepOnePlus Real-Time PCR Systems (Applied Biosystems) as follows: 2 µL of IMP-1 enzyme (100 µM), 1 µL of EDTA or HHIA solution (5 mM), 2.5 µL of diluted Protein Thermal Shift Dye (32×), 5 µL of Protein Thermal Shift buffer, and 9.5 µL of water were mixed in a test tube and incubated for 15 min at 4°C. Four replicates of each sample were performed. The fluorescence was monitored at 0.7°C increments from 4°C to 99°C. The relative fluorescence units were fitted with the Boltzmann equation using the TSA-CRAFT software to determine the melting point (*T*_*m*_) (47).

### Zinc quantitative assay

A total of 5 µL of IMP-1 (100 µM) and 2 µL of EDTA (5 mM) or HHIA (5 mM) were mixed in 100 µL of 20 mM HEPES buffer (pH 7.5) pretreated with Chelex 100 resin (Bio-Rad) and incubated on ice for 30 min. The samples were dialyzed against ultrapure water at 4°C for 6 h using Slide-A-Lyzer Dialysis Cassettes (cutoff, 3.5 K) (Thermo Fisher Scientific) and treated with proteinase K (FUJIFILM Wako Pure Chemical Corporation) at 37°C for 1 h. The zinc concentration in the samples was determined using a Zinc Assay Kit (MG Metallogenics).

### X-ray crystallography

The purified IMP-1 (60 mg/mL) was mixed with an equal volume reservoir solution [0.2 M sodium acetate, 0.1 M HEPES-NaOH (pH 7.7), and 35% polyethylene glycol 3350] and crystallized with sitting-vapor diffusion method. The IMP-1 crystals were clashed and subjected to microseeding. The crystals obtained after microseeding were soaked in a reservoir solution containing 10-HHIA before collecting X-ray diffraction data. The purified VIM-2 (15 mg/mL) was mixed with an equal volume reservoir solution (0.2 M magnesium formate and 25% polyethylene glycol 3350) and crystallized. The crystals were soaked in a reservoir solution containing 10-HHIA before collecting X-ray diffraction data. The X-ray diffraction data were collected at the BL-5A beamline (Photon Factory, Ibaraki, Japan) and the BL2S1 beamline (Aichi Synchrotron Radiation Center, Aichi, Japan). Diffraction data were processed using iMosflm (48) in the CCP4 suite (49). The crystal structure was solved via molecular replacement using the MOLREP program (50) in the CCP4 suite (49). Model building was performed using COOT (51), and model refinement was performed using REFMAC5 (52) in the CCP4 suite (49).

### Docking simulation

Refinement of the IMP-1 crystal structure (PDB ID: 5Y5B) was performed using Homology Modeling Professional for HyperChem software (53–55). The calculations were made using Amber99 force field with the following parameters: RMS gradient, 1.0 kcal mol^−1^ Å^−1^; algorithm, Polak-Ribière; cutoffs, none; 1–4 van der Waals scale factor, 0.5; 1–4 electrostatic scale factor, 0.833; dielectric scale factor, 1.0; and dielectric condition, distance dependent. Biomacromolecule-rigid and ligand-flexible docking simulations of (*S*)10-HHIA for the refined IMP-1 structure, wherein all water molecules and sulfonic acid in the active site were previously removed, were performed using the Docking Study with HyperChem (DSHC) software (53, 56) under the above calculation conditions to obtain the most stable docking mode. The detailed procedure and algorithm of DSHC have been previously reported (57).

## Data Availability

Atomic coordinates and structural factors of the VIM-2–10-HHIA complex were deposited in the Protein Data Bank database and controlled under accession number PDB ID 8I52. The whole genome sequence data of *Aspergillus* sp. OPMF00815 strain was deposited in the DNA Data Bank of Japan (DDBJ) database and is controlled under accession number BTWD01000001–BTWD01001181.

## SUPPLEMENTAL MATERIAL

Text S1 spectrum.02344-23-s0001.docx Supplemental text.

Fig. S1 spectrum.02344-23-s0002.tif Results of HPLC and LC-MS analyses.

Fig. S2 spectrum.02344-23-s0003.tif 13C-NMR data.

Fig. S3 spectrum.02344-23-s0004.tif 1H-NMR data.

Fig. S4 spectrum.02344-23-s0005.tif Picture and tree view.

Fig. S5 spectrum.02344-23-s0006.tif Electron density map of 10-HHIA.

Table S1 spectrum.02344-23-s0007.docx MIC data.

Table S2 spectrum.02344-23-s0008.docx Crystallography data.

ASM does not own the copyrights to Supplemental Material that may be linked to, or accessed through, an article. The authors have granted ASM a non-exclusive, world-wide license to publish the Supplemental Material files. Please contact the corresponding author directly for reuse.
