# Supplementary material for: Hydroxyhexylitaconic acids as potent IMP-type metallo-β-lactamase inhibitors for controlling carbapenem resistance in Enterobacterales
Source: Microbiol Spectr. 2024 Feb 5;12(3):e02344-23. doi: 10.1128/spectrum.02344-23 (PMC10913484; doi:10.1128/spectrum.02344-23)
Supplement: Text S1 — Supplemental text. [file spectrum.02344-23-s0001.docx]

**Supplemental text 1.**

**Supplemental methods**

**Cell-based screening.** The extract libraries, 5488 samples of microorganisms (mainly *Actinomycetales* and filamentous fungi) provided by OP Bio Factory (Okinawa, Japan), were screened against *E. coli* DH5α expressing IMP-1 (MPM MIC, 1 μg/mL). The *bla*_IMP-1_ gene and its promoter region was amplified by PCR, ligated to pBC-SK(+) phagemid, and transformed into *E. coli* DH5α in the previous work (1). Mueller-Hinton II (MH-II) broth containing *E. coli* DH5α expressing IMP-1 was inoculated into 96-well microtiter plates containing MH-II broth supplemented with MPM (1/8 MIC, 0.125 μg/mL) and an aliquot of microorganism samples. The plates were incubated at 35°C for 18 h, and the wells showing no visible bacterial growth were initially selected. Separately, the original toxicity of the samples toward live bacterial cells was investigated using an indicator of bacterial growth in MH-II broth containing the sample alone. Finally, samples showing growth inhibition under MPM without any original toxicity were selected.

**Enzyme-based screening.** Purified IMP-1 (10 nM) and an aliquot of the selected samples were incubated in 20 mM HEPES buffer (pH 7.5) containing 200 mM NaCl and 50 μg/mL bovine serum albumin (BSA) at 30°C for 5 min. Imipenem was added as a substrate to attain 100 μM, and the behavior of Abs_297_ was monitored using a spectrophotometer UV-2700 (Shimadzu) at 30°C. The residual activity was defined as follows: ([Δabs_297_/min] with samples)/([Δabs_297_/min] without samples)] × 100 (%)

**Preliminary experiments for extraction and purification of IMP-1 inhibitory components in *Aspergillus* sp. OPMF00815.** The *Aspergillus* sp. OPMF00815, grown on LCA agar plates, was inoculated in modified Potato Dextrose Broth (mPDB) [PDB with KNO_3_, KH_2_PO_4_, Mg_2_SO_4_, and artificial sea water], and incubated at 28°C for 7 days at 200 rpm. Aliquots of pre-cultured cells were further inoculated into fresh mPDB and incubated in a shaker at 28°C for 7 days at 200 rpm. The culture broth was centrifuged at 3,000 rpm for 5 min. The pelleted mycelia were then treated with acetone and methanol. The organic solvent layer was condensed and mixed with the culture medium supernatant. This mixture was treated twice with ethyl acetate and the ethyl acetate layer was collected and condensed. The extracts were dissolved in DMSO and subjected to high-performance liquid chromatography (HPLC) (JASCO) using a Pegasil ODS SP100 column (20 × 150 mm) (BGB Analytik). The mobile phase consisted of solvent A (0.1% formic acid-acetonitrile) and solvent B (methanol). Elution was performed with an increased gradient of 5% to 100% solvent B in 0 to 35 min and 100% solvent B in 35 to 40 min. The flow rate was 10 mL/min, and the eluted fraction was collected every 1 min (10 mL per collection tube) and monitored at 210 nm. The eluted samples in each collection tube were subjected to cell-based and enzyme-based assays to confirm their inhibitory activity. The samples showing IMP-1 inhibitory activities were subjected to UPLC-MS analysis using an ACQUITY UPLC (Waters) equipped with an ACQUITY UPLC BEH C18 column (2.1 × 50 mm) (Waters) and Xevo G2-S QTOF systems (Waters). The mobile phase consisted of solvent A (0.1% formic acid) and solvent B (0.1% formic acid-acetonitrile). The elution was performed with an increased gradient of 5% to 100% solvent B for 0 to 5 min. The elution rate was 0.8 mL/min.

**Next-generation sequencing.** The *Aspergillus* sp. strain OPMF00815 provided by OP Bio Factory, grown on LCA agar plates, was inoculated in mPDB and incubated at 28°C for 7 days at 180 rpm. The cultured broth was filtered through Whatman paper to collect mycelia. The mycelia were washed with sterile water, frozen in liquid nitrogen, and crushed using a pestle and mortar. The solution (50 mM EDTA [pH8.0], 0.5% SDS, and 50 mg/mL Proteinase K) containing crushed mycelia was incubated at 60°C for 1 h. An equal volume of phenol/chloroform/isoamyl alcohol (25:24:1) was then added, followed by thorough mixing, and the mixture was then centrifuged. After centrifugation, the aqueous phase was collected and mixed with an equal volume of chloroform/isoamyl alcohol (24:1). After centrifugation, the aqueous phase was collected and precipitated using sodium acetate and anhydrous ethanol. The precipitant was resuspended in the ATL buffer of the QIAamp DNA Mini Kit (Qiagen) and subsequently processed according to the manufacturer’s protocol. The DNA concentration was determined using a Qubit 4 fluorometer (Thermo Fisher Scientific). The NEBNext Ultra DNA Library Prep Kit (New England Biolabs) was used for library preparation. The DNA libraries were sequenced using the NovaSeq 6000 platform (Illumina). De novo assembly was performed using CLC Genomics Workbench (Qiagen) with default parameters.

A tree (a neighbor-joining tree with Tamura-Nei distances) showing phylogenetic relationships was drawn based on the rDNA-ITS, β-tubulin, and calmodulin combined sequences using MEGA11 software (2). The sequences needed to construct the tree were obtained from a previous report (3). Pairwise average nucleotide identity (ANI) values were calculated using an ANI calculator (4).

**NMR**

**9-HHIA (compound A):** ^1^H NMR (600 MHz, CD_3_OD) *δ* 6.31 (δ, *J* = 0.54 Hz, 1H), 5.74 (δ, *J* = 0.54 Hz, 1H), 3.71–3.64 (m, 1H), 3.43 (t, *J* = 7.2 Hz, 1H), 1.87–1.81 (m, 1H), 1.70–1.62 (m, 1H), 1.46–1.26 (m, 6H), 1.12 (δ, *J* = 6.2 Hz, 3H); ^13^C NMR (150 MHz, CD_3_OD) *δ* 177.1, 169.5, 140.9, 127.0, 68.5, 48.0, 40.0, 32.3, 28.8, 26.7, 23.5.

**10-HHIA (compound B):** ^1^H NMR (600 MHz, CD_3_OD) *δ* 6.34 (δ, *J* = 0.54 Hz, 1H), 5.77 (δ, *J* = 0.54 Hz, 1H), 3.51 (t, *J* = 6.6 Hz, 2H), 3.43 (t, *J* = 7.2 Hz, 1H), 1.87–1.80 (m, 1H), 1.70–1.61 (m, 1H), 1.54–1.47 (m, 2H), 1.33 (m, 6H); ^13^C NMR (150 MHz, CD_3_OD) *d* 177.1, 169.5, 140.9, 127.0, 63.0, 48.0, 33.6, 32.2, 30.3, 28.7, 26.8.

**References**

1. Wachino JI, Jin W, Kimura K, Kurosaki H, Sato A, Arakawa Y. 2020. Sulfamoyl heteroarylcarboxylic acids as promising metallo-β-lactamase inhibitors for controlling bacterial carbapenem resistance. mBio. 11(2):doi:10.1128/mBio.03144-19.

2. Tamura K, Stecher G, Kumar S. 2021. MEGA11: Molecular Evolutionary Genetics Analysis Version 11. Mol Biol Evol 38:3022-3027. doi:10.1093/molbev/msab120.

3. Hong SB, Lee M, Kim DH, Varga J, Frisvad JC, Perrone G, Gomi K, Yamada O, Machida M, Houbraken J, Samson RA. 2013. *Aspergillus luchuensis*, an industrially important black *Aspergillus* in East Asia. PLoS One 8:e63769. doi:10.1371/journal.pone.0063769.

4. Goris J, Konstantinidis KT, Klappenbach JA, Coenye T, Vandamme P, Tiedje JM. 2007. DNA-DNA hybridization values and their relationship to whole-genome sequence similarities. Int J Syst Evol Microbiol 57:81-91. doi:10.1099/ijs.0.64483-0.
