# Supplementary figures and images for: Hydroxyhexylitaconic acids as potent IMP-type metallo-β-lactamase inhibitors for controlling carbapenem resistance in Enterobacterales
Source: Microbiol Spectr. 2024 Feb 5;12(3):e02344-23. doi: 10.1128/spectrum.02344-23 (PMC10913484; doi:10.1128/spectrum.02344-23)

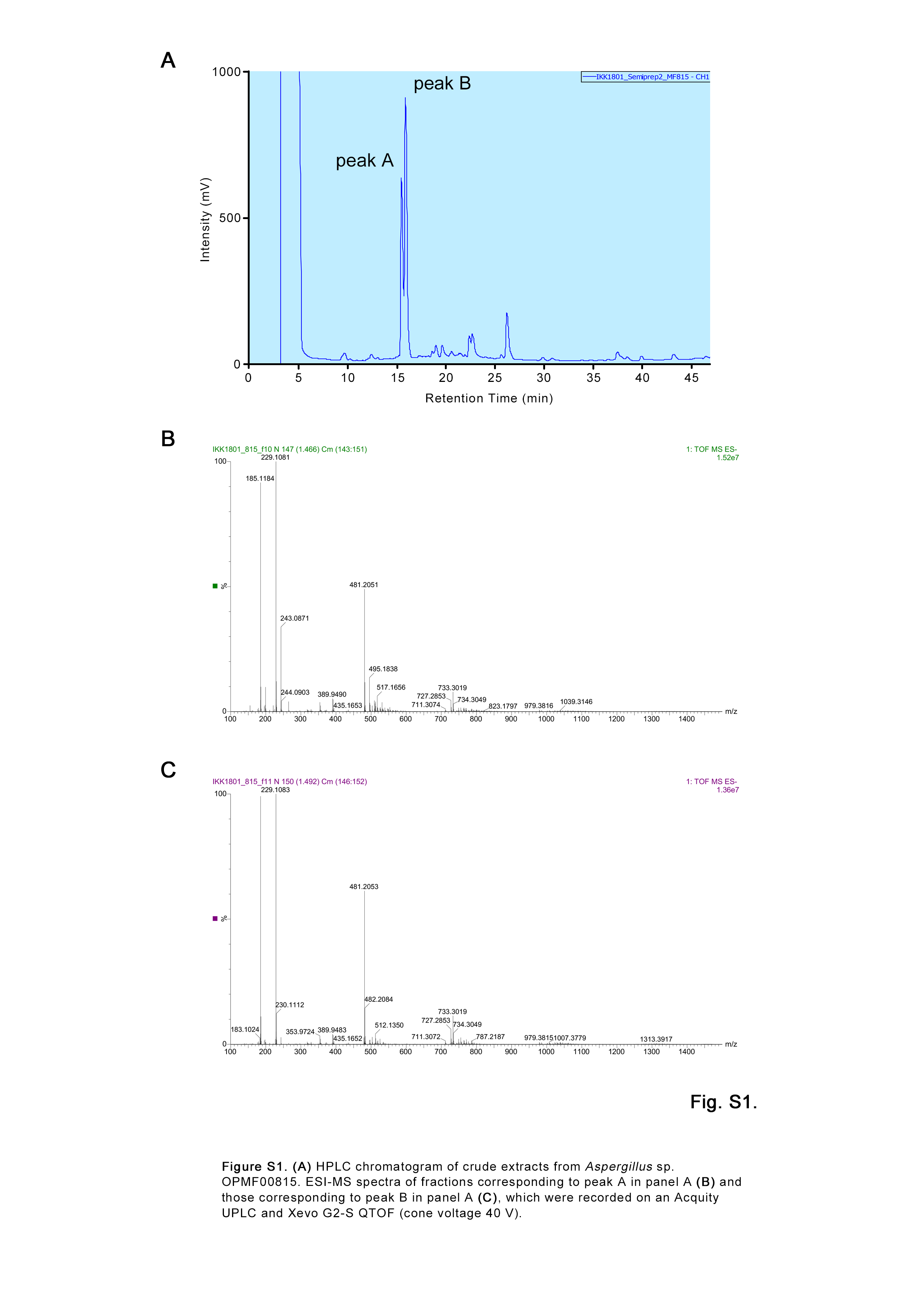

Supplement: Fig. S1 — Results of HPLC and LC-MS analyses. [file spectrum.02344-23-s0002.tif]

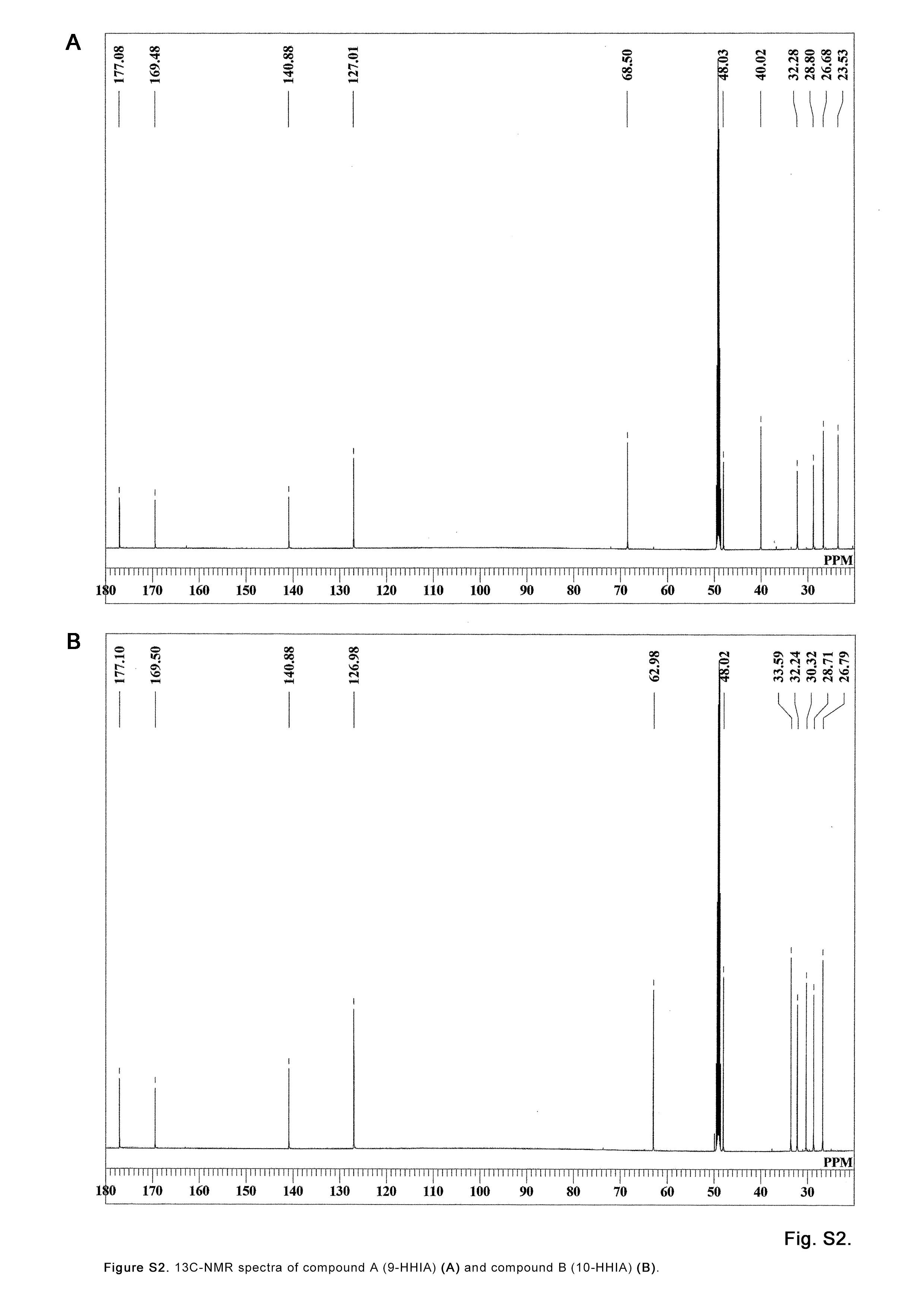

Supplement: Fig. S2 — 13C-NMR data. [file spectrum.02344-23-s0003.tif]

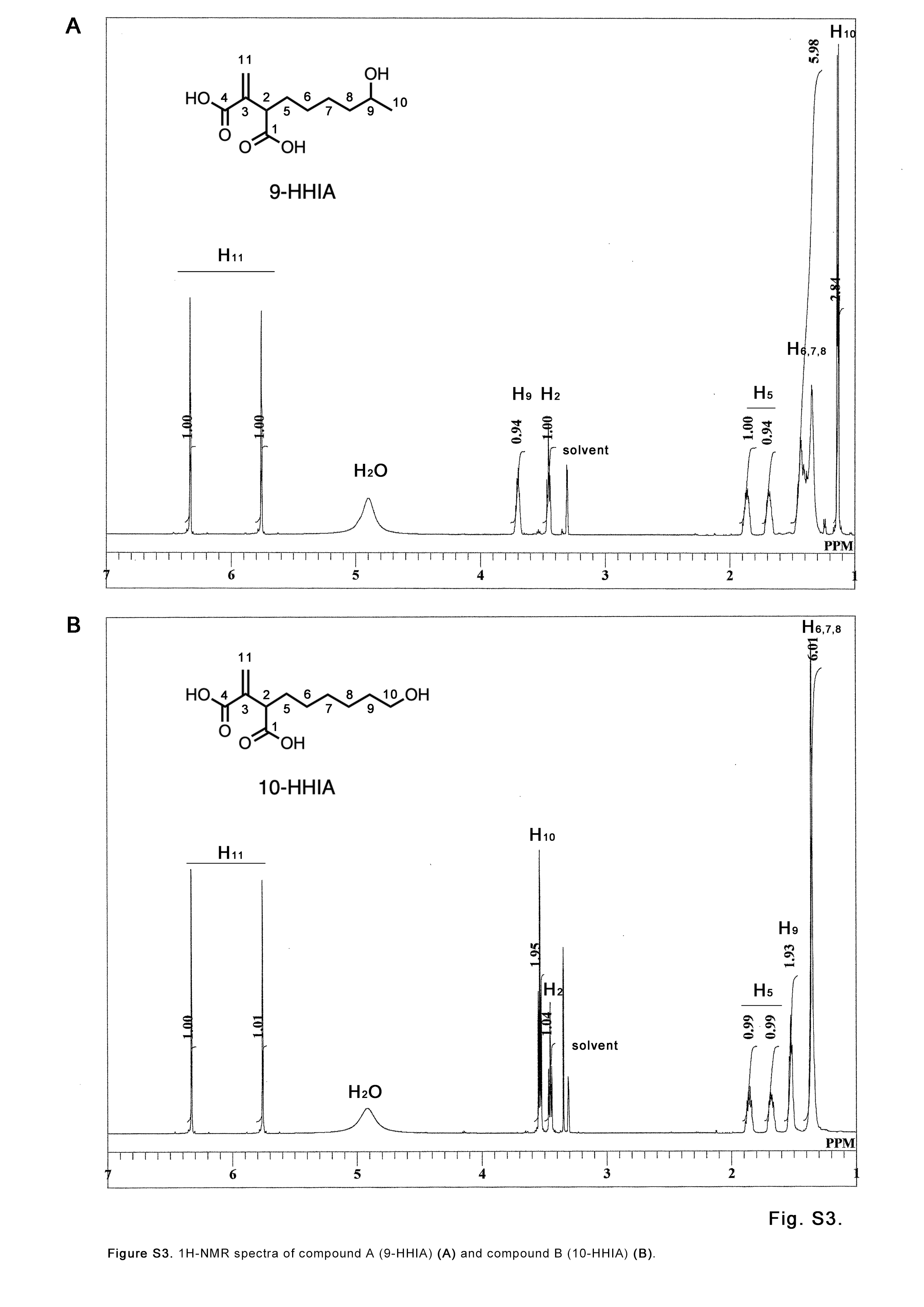

Supplement: Fig. S3 — 1H-NMR data. [file spectrum.02344-23-s0004.tif]

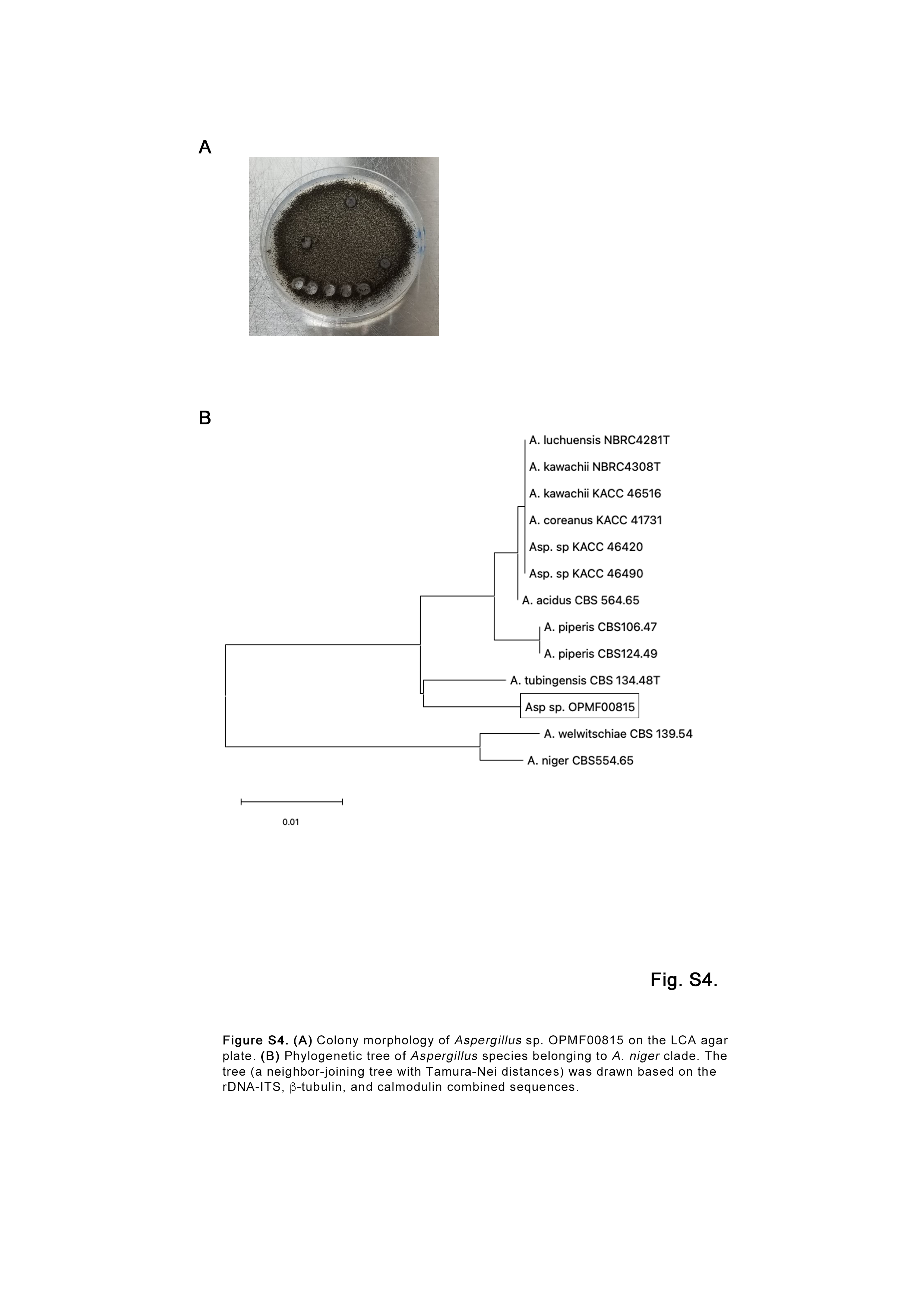

Supplement: Fig. S4 — Picture and tree view. [file spectrum.02344-23-s0005.tif]

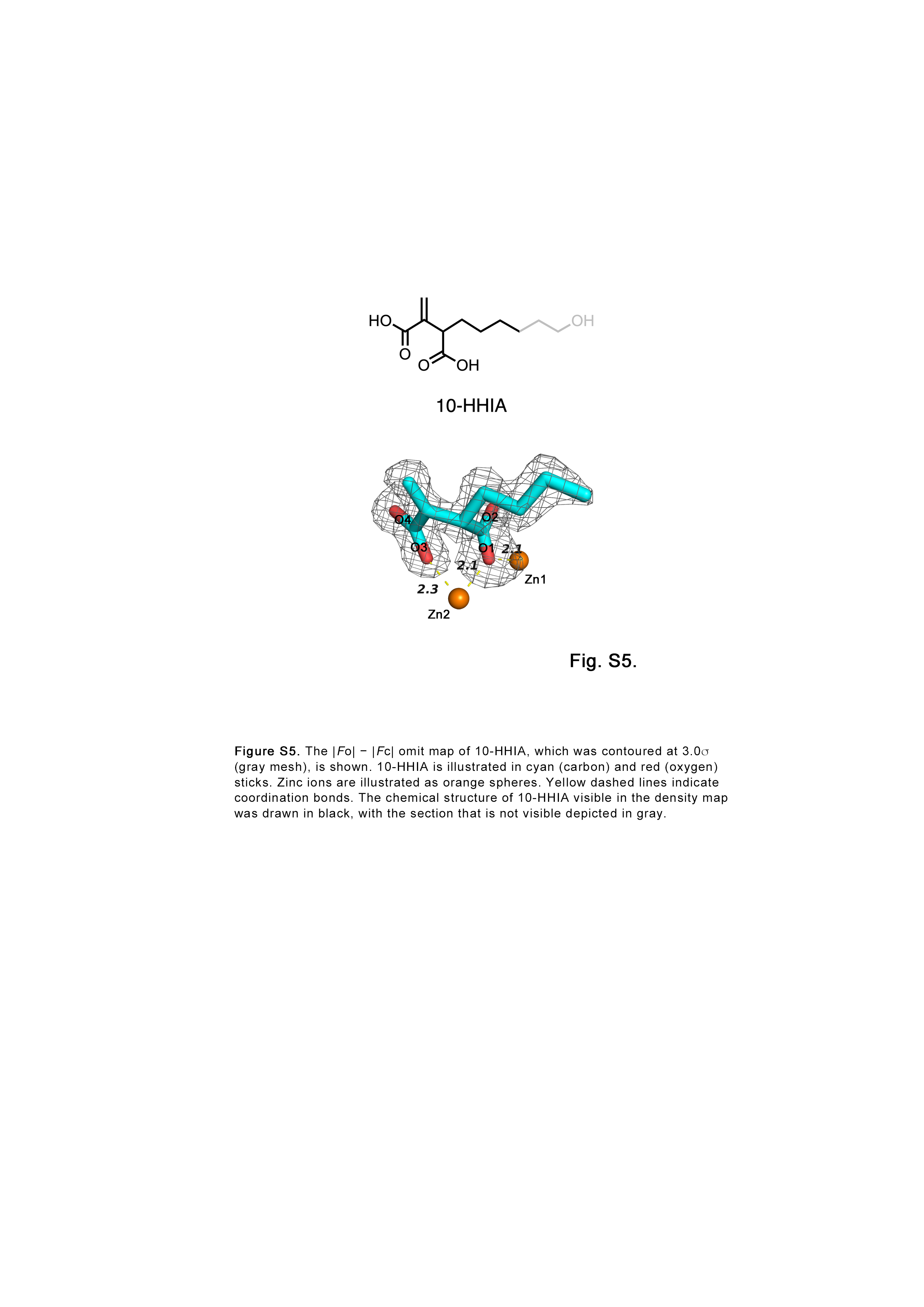

Supplement: Fig. S5 — Electron density map of 10-HHIA. [file spectrum.02344-23-s0006.tif]
