# Supplementary material for: Hydroxyhexylitaconic acids as potent IMP-type metallo-β-lactamase inhibitors for controlling carbapenem resistance in Enterobacterales
Source: Microbiol Spectr. 2024 Feb 5;12(3):e02344-23. doi: 10.1128/spectrum.02344-23 (PMC10913484; doi:10.1128/spectrum.02344-23)
Supplement: Table S1 — MIC data. [file spectrum.02344-23-s0007.docx]

| **Supplemental table S1**. Results of susceptibility tests for MBL-producing *Enterobacterales* isolates. | | | | | | | | | | |
| --- | --- | --- | --- | --- | --- | --- | --- | --- | --- | --- |
|  |  | IPM MIC (μg/mL) | | | | | | | | |
| Bacterial strains | MBLs | Control |  | +9-HHIA | | |  | +10-HHIA | | |
|  |  |  |  | (6.25)* | (25)* | (100)* |  | (6.25)* | (25)* | (100)* |
| *E. coli* NUBL-22 | IMP-6 | 2 |  | 2 | 1 | 0.5 |  | 1 | 0.5 | 0.5 |
| *E. coli* NUBL-24 | IMP-1 | 8 |  | 4 | 2 | 1 |  | 4 | 1 | 1 |
| *E. coli* 426 | IMP-1 | 8 |  | 8 | 4 | 2 |  | 8 | 4 | 2 |
| *E. coli* 465 | IMP-1 | 32 |  | 16 | 8 | 2 |  | 16 | 4 | 1 |
| *K. pneumoniae* NUBL-8 | IMP-1 | 4 |  | 2 | 4 | 1 |  | 2 | 1 | 0.5 |
| *K. pneumoniae* AR0034 | IMP-4 | 4 |  | 4 | 2 | 0.5 |  | 2 | 1 | 0.5 |
| *K. pneumoniae* NUBL-23 | IMP-6 | 1 |  | 0.5 | 0.5 | 0.5 |  | 0.5 | 0.25 | 0.5 |
| *K. oxytoca* NUBL-827 | IMP-1 | 16 |  | 8 | 4 | 2 |  | 8 | 4 | 2 |
| *P. penneri* E11-M475 | IMP-1 | >64 |  | >64 | >64 | >64 |  | >64 | >64 | 64 |
| *E. cloacae* NUBL-5 | IMP-1 | 8 |  | 8 | 4 | 2 |  | 8 | 4 | 1 |
| *K. pneumoniae* MS5674 | NDM-1/VIM-1 | 32 |  | 64 | 32 | 32 |  | 32 | 64 | 32 |
| *K. pneumoniae* AR0076 | VIM-1 | 32 |  | 32 | 32 | 32 |  | 32 | 32 | 16 |
| *, μg/mL |  |  |  |  |  |  |  |  |  |  |
|  |  |  |  |  |  |  |  |  |  |  |
