# Supplementary material for: Hydroxyhexylitaconic acids as potent IMP-type metallo-β-lactamase inhibitors for controlling carbapenem resistance in Enterobacterales
Source: Microbiol Spectr. 2024 Feb 5;12(3):e02344-23. doi: 10.1128/spectrum.02344-23 (PMC10913484; doi:10.1128/spectrum.02344-23)
Supplement: Table S2 — Crystallography data. [file spectrum.02344-23-s0008.docx]

| **Supplemental table 2.** Data collection and refinement statistics | |
| --- | --- |
| Data collection |  |
| Beam line | PF BL-5A |
| Wavelength (Å) | 1.0000 |
| Resolution range (Å) | 55.4 − 1.58 (1.67− 1.58) |
| Space group | *C*2 |
| Cell dimensions |  |
| *a* (Å) | 101.3 |
| *b* (Å) | 79.3 |
| *c* (Å) | 67.9 |
| α (°) | 90.0 |
| β (°) | 130.1 |
| γ (°) | 90.0 |
| No. of unique reflections | 55926 (8043) |
| Redundancy | 6.6 (6.7) |
| Completeness (%) | 99.7 (98.7) |
| *R*_merge_ (%) | 8.2 (20.4) |
| mean *I*/*σ* (*I*) | 16.0 (8.0) |
|  |  |
| Refinement |  |
| *R*_working_ (%) | 0.147 |
| *R*_free_ (%) | 0.179 |
| r.m.s.d. |  |
| Bond lengths (Å) | 0.012 |
| Bond angles (°) | 1.704 |
